# The associations of ferritin, serum lipid and plasma glucose levels across pregnancy in women with gestational diabetes mellitus and newborn birth weight

**DOI:** 10.1186/s12884-023-05806-z

**Published:** 2023-06-29

**Authors:** Jing Ji, Pei Wu, Guohua Li, Zhangya He, Shanshan Wang, Wenlu Yu, Chao Li, Yang Mi, Xiaoqin Luo

**Affiliations:** 1 Department of Nutrition and Food Safety, School of Public Health, Xi’an Jiaotong University, Xi’an, 710061 China; 2 Department of Obstetrics and Gynecology, Northwest Women’s and Children’s Hospital, Xi’an, 710061 China; 3 Key Laboratory of Population Health Across Life Cycle of Ministry of Education, Hefei, China; 4 Department of Epidemiology and Health Statistics, School of Public Health, Xi’an Jiaotong University, Xi’an, 710061 China

**Keywords:** Gestational diabetes mellitus, Ferritin, Lipid profile, Fasting plasma glucose, Birth weight

## Abstract

**Background:**

Women with gestational diabetes mellitus (GDM) are at greater risk of abnormal birth weight. Since the level of biochemical indicators could often affect the intrauterine growth and development of the fetus, it is of great practical significance to understand the changes of biochemical levels across pregnancy in women with GDM and to find out the indicators that play an important role in predicting birth weight.

**Methods:**

The data source of this study was from the Xi'an Longitudinal Mother–Child Cohort study (XAMC), in which women with GDM with normal and high pre-pregnancy body mass index (BMI) and their newborns between January 1^st^ and March 31^st^ in 2018 were included. The data of ferritin, serum lipid profile and fasting plasma glucose (FPG) of mothers in the three trimesters of pregnancy, as well as birth weight of newborns were all collected from medical records. Multiple linear regression and multivariate logistic regression analyses were used to explore the association of the biochemical indexes and birth weight. A *P* value < 0.05 was considered statistically significant.

**Results:**

A total of 782 mother-infant pairs were finally included and divided into normal weight group (NG) (*n* = 530, 67.8%) and overweight/obesity group (OG) (*n* = 252, 32.2%) according to maternal pre-pregnancy BMI. The level of ferritin in both NG and OG decreased during pregnancy (*P* for trend < 0.001 for all), whereas the levels of total cholesterol (TC), high density cholesterol (HDL-C), low density cholesterol (LDL-C) and triglycerides (TG) all showed an upward trend (*P* for trend < 0.05 for all). The levels of FPG in the two groups remained in a relatively stable during the whole pregnancy even though it was higher in OG during the 2^nd^ and 3^rd^ trimesters, whilst HbAlc levels in NG women increased (*P* for trend = 0.043) during pregnancy. Meanwhile, the risk of macrosomia and large-for-gestational-age (LGA) increased with the increase of FPG level (*P* for trend < 0.05). Multivariate logistic regression analyses results showed that only FPG level in the 3^rd^ trimester was correlated with birth weight, with birth weight increased by 44.9 g for each SD increase in FPG level.

**Conclusion:**

Maternal FPG in the 3^rd^ trimester is an independent predictor of newborn birth weight, and a higher level of that is associated with an increased risk of macrosomia and LGA.

## Background

Gestational diabetes mellitus (GDM) is defined as "diabetes diagnosed in the second or third trimester of pregnancy that is not clearly overt diabetes prior to gestation" [1], is one of the most common metabolic diseases during pregnancy. The incidence has been on the rise in recent decades, although there are significant differences globally [2]. GDM could lead to adverse maternal and neonatal outcomes including shoulder dystocia, stillbirth, metabolic diseases in childhood and adolescence, etc [3–5]. The effect of GDM on birth weight is a matter of concern as it could increase the risk of abnormal birth weight [6]. Hence, there is an urgent need for effective measures for prenatal intervention.

The level of maternal biochemical indicators could often reflect the health of the mother during pregnancy and their offspring. It is well known that birth weight is associated with maternal metabolic disturbances, such as hyperglycemia, dyslipidemia, and other health problems like high iron content which is suggested as the level of ferritin. Hence, it is helpful to predict and monitor the birth weight through the detection of those clinical biochemical indicator, such as ferritin, serum lipid profile, plasma glucose and glycosylated hemoglobin (HbAlc), and some of them were well documented in normal pregnant women [7–9]. Since the level of maternal biochemical indexes change as pregnancy progresses [10], their levels in different stages of pregnancy may have various predictive effects on birth weight of newborns. Hence, it is not enough to only focused on the level of biochemical index in a certain stage of pregnancy [11–13]. Until now, the occurrence of altered serum ferritin, lipid profile and metrics of sugar metabolism have not been fully elucidated in women with GDM. Therefore, the present investigation was carried out to assess the changes of some important biochemical indexes, such as ferritin, serum lipid profile and glucose level in the different trimesters of pregnancy in women with GDM and their relationship with birth weight.

## Methods

### Data source and study population

The data source of this study was from the Xi'an longitudinal mother–child cohort (XAMC), which is an ongoing large-scale longitudinal mother–child cohort study to investigate the role of external and internal factors on the short-term and long-term outcomes of mothers and children [14]. All pregnant women delivered at Northwest Women's and Children's Hospital between January 1^st^ and March 31^st^ in 2018 were included. From the medical records, we excluded subjects with type 1 or 2 diabetes mellitus before pregnancy as well as those who were not diagnosed with GDM. Patients with multiple birth, incomplete or incorrect data, and stillbirth, or women with a history of severe systemic disease such as cancer, chronic renal failure, severe anemia, or immune disorders and other endocrinopathies were also excluded. Only women with GDM with normal and high pre-pregnancy BMI who had more than one of biochemical index records were selected.

The data of maternal age, parity, gestational age, admission/discharge diagnosis, sociodemographic information, anthropometric characteristics of mothers and newborns, maternal biochemical parameters including ferritin, lipid profile, such as total cholesterol (TC), high-density lipoprotein cholesterol (HDL-C), low-density lipoprotein cholesterol (LDL-C), triglyceride (TG), as well as fasting plasma glucose (FPG) and HbAlc were all extracted from the electronic information system of hospital. Generally, the biochemical parameters were measured in the first (gestational age < 13 weeks), middle (13–28 gestational weeks) and third (> 28 weeks) trimesters of pregnancy.

The protocol was approved by the ethical committee of Xi'an Jiaotong University (XJTU 2016–053) and the Northwest Women's and Children's Hospital (NWCH 2012–013). All patients provided written informed consent. The principles of the Helsinki Declaration were followed throughout the study.

### GDM diagnosis

All women underwent a 75-g oral glucose tolerance test (OGTT) during 24–28 gestational weeks. The diagnosis was carried out by obstetricians based on the recommendations of the International Association of Diabetes and Pregnancy Study Groups Consensus Panel [15]: fasting glucose 5.1 mmol/L, 1-h glucose 10.0 mmol/L, or 2-h glucose 8.5 mmol/L. The plasma glucose greater than or equal to any of these thresholds was diagnosed as GDM.

### Classification and definition

Pre-pregnancy BMI (weight (kg)/ height (m)^2^) was calculated by maternal height and pre-pregnancy weight. Mothers were divided into two groups according to pre-pregnancy BMI: Normal weight group (NG, BMI:18.5–23.9 kg/m^2^) and Overweight/Obese group (OG, BMI ≥ 24.0 kg/m^2^) [16]. Low birth weight (LBW) was defined as birth weight less than 2500 g whilst macrosomia was defined as over 4000 g after 37 weeks' gestation. Infants whose fetal growth less than the 10^th^ percentile at each completed week of gestation were small for gestational age (SGA), and greater than 90^th^ percentile were large for gestational age (LGA) [17]. For educational level, "senior high and below", "undergraduate", and "bachelor above" were regarded as "low", "middle", and "high", respectively.

### Statistical analysis

Continuous measures were expressed as mean and standard deviation (SD), while discrete variables were expressed as number and percentage. For continuous variables with normal distribution, Independent-Samples *T* Test was used for intergroup comparison, whilst *Kruskal–Wallis H* test was used for those with non-normal distribution. The *Chi*-square test (Fisher exact test) was used for categorical variables. Trend tests were used to evaluate the changing trends of the data. In multiple linear regression, the biochemical variables were standardized by Z-score method which was implemented by SPSS, and the linear relationships between biochemical indexes and birth weight were analyzed and presented as *β* (95% *CI*). Moreover, the relationships between the level of biochemical indexes and the risk of macrosomia and LGA were analyzed by logistic regression analyses and presented as OR (95% *CI*). All the analyses were performed by the statistical package SPSS (version 23.0, Chicago, IL), and GraphPad Prism 8.0.1 was used for drawing. *P*-value less than 0.05 was considered statistically significant.

## Results

### Characteristics of mother-infant pairs

A total of 782 mother-infant pairs were included and 67.8% (*n* = 530) of them had normal pre-pregnancy BMI (Table 1). The average age of mothers at birth was 31.9 ± 4.2 years old, and 71.1% of mothers had medium education level. More than sixty percent of mothers were uniparas, and 1/10 of mothers had a family history of diabetes. The gestational weight gain in OG women was lower than that in NG (10.6 ± 4.5 kg *vs* 13.2 ± 4.7 kg, *P* < 0.001). Compared with NG, the proportion of preterm delivery and cesarean section in OG was much higher (7.5% *vs* 3.6%, *P* < 0.05; 61.1% *vs* 44.9%, *P* < 0.001). For newborns, the average birth weight was 3405.6 ± 460.7 g, and that is higher in OG although it was just marginally significant (3382.6 ± 429.6 g *vs* 3454.1 ± 517.6 g, *P* = 0.058). Moreover, both the ratios of LBW and LGA in OG were higher than those in NG (5.6% vs 1.9%; 17.1% vs 10.9%, *P* < 0.05 for all).

**Table 1:** Baseline characteristics and neonatal outcomes of women with gestational diabetes mellitus

	Total (*n* = 782)	NG (*n* = 530)	OG (*n* = 252)	*P*
Maternal characteristics				
Age (years)	31.9 ± 4.2	31.8 ± 4.2	32.3 ± 4.1	0.097
Educational level (%)				0.279
Low	113(14.5)	72(13.6)	41(16.3)	
Middle	556(71.1)	375(70.7)	181(71.8)	
High	113(14.4)	83(15.7)	30(11.9)	
Preterm (%)	38(4.9)	19(3.6)	19(7.5)	0.016
Gestational weight gain (kg)	12.4 ± 4.8	13.2 ± 4.7	10.6 ± 4.5	< 0.001
Parity (%)				0.294
0	480(61.4)	332(62.6)	148(58.7)	
≥ 1	302(38.6)	198(37.4)	104(41.3)	
Birth delivery (%)				< 0.001
Vaginal delivery	390(49.9)	292(55.1)	98(38.9)	
Cesarean section	392(50.1)	238(44.9)	154(61.1)	
Family history of diabetes (%)	79(10.1)	51(9.6)	28(11.1)	0.519
Neonatal outcomes				
Birth weight (g)	3405.6 ± 460.7	3382.6 ± 429.6	3454.1 ± 517.6	0.058
LBW (%)	24(3.1)	10(1.9)	14(5.6)	0.005
SGA (%)	22(2.8)	19(3.6)	3(1.2)	0.058
Macrosomia (%)	75(9.6)	44(8.3)	31(12.3)	0.076
LGA (%)	101(12.9)	58(10.9)	43(17.1)	0.017
Sex (%)				0.918
Male	421(53.8)	286(54.0)	135(53.6)	
Female	361(46.2)	244(46.0)	117(46.4)	

*NG* Normal Group, *OG* Overweight/obese Group

*LBW* Low Birth Weight (birth weight < 2500 g); Macrosomia (birth weight ≥ 4000 g), *SGA* Small for Gestational Age (birth weight less than the 10^th^ percentile at each completed week of gestation), *LGA* Large for Gestational Age (birth weight greater than 90^th^ percentileat each completed week of gestation)

For educational level, "senior high and below", "undergraduate", and "bachelor above" were regarded as "low", "middle", and "high", respectively

*P* < 0.05 means there was a statistical difference between the two groups

### Trends of biochemical indexes’ levels during pregnancy

**Fig. 1:**
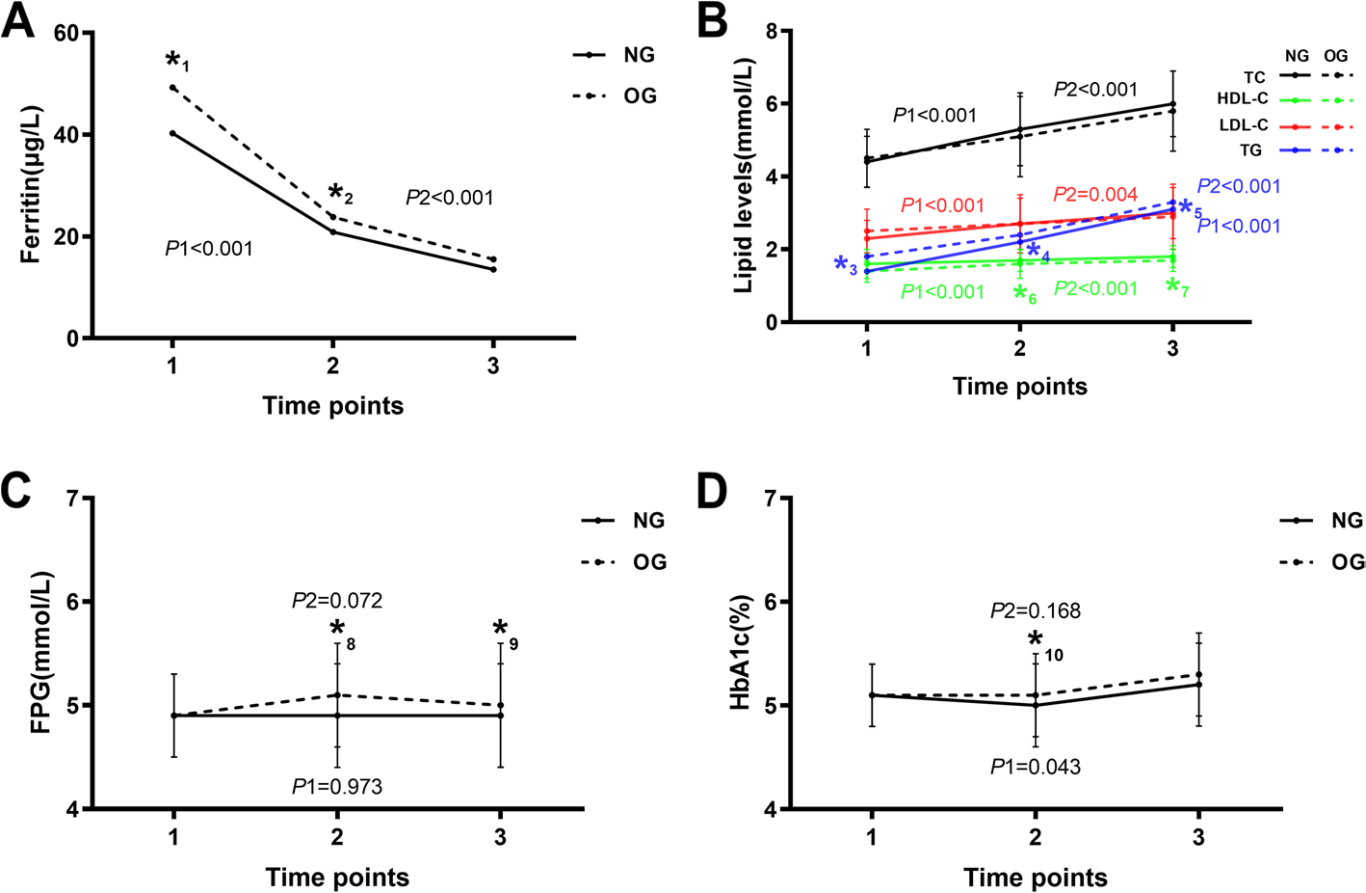
Level changes of **a** ferritin, **b** lipid, **c** FPG and **d** HbAlc across pregnancy in women with GDM. NG: Normal Group; OG: Overweight/obese Group; TC: total cholesterol; HDL-C: high-density lipoprotein cholesterol; LDL-C: low-density lipoprotein cholesterol; TG: triglyceride; FPG: fasting plasma glucose. 1: The 1^st^ trimester; 2: The 2^nd^ trimester; 3: The 3^rd^ trimester. *: There was a statistical difference between the two groups at this time point. *P* values: *_1_: 0.013; *_2_:0.042; *_3_:0.021; *_4_:0.047; *_5_: 0.035; *_6_: 0.030; *_7_: 0.034; *_8_: 0.019; *_9_: 0.044; *_10_: 0.038. *P*1: *P* for trend in NG. *P*2: *P* for trend in OG

We analyzed the changing trend of the average level of biochemical indexes in different trimesters in NG and OG, respectively (Fig. 1). The results showed statistically significant reduction in ferritin concentration in both groups (*P* for trend < 0.001 for all). Nevertheless, compared with NG, the level of ferritin in OG was remarkably higher in the 1^st^ and 2^nd^ trimesters [49.3 (32.7, 70.3) μg/L vs 40.3 (23.2, 65.5) μg/L, *P* < 0.05; 23.8 (13.5, 47.7) μg/L vs 20.9 (11.6, 36.7) μg/L, *P* < 0.05]. Contrarily, lipid profile data showed pronounced elevations of TC, HDL-C, LDL-C and TG during the whole pregnancy (*P* for trend < 0.05 for all). Moreover, in the 2^nd^ and 3^rd^ trimester, the HDL-C level of NG was higher than that of OG [(1.7 ± 0.3) mmol/L vs (1.6 ± 0.4) mmol/L; (1.8 ± 0.3) mmol/L vs (1.7 ± 0.3) mmol/L, *P* < 0.05 for all], while the TG level of OG was significantly higher than that of NG during the whole pregnancy [1.8 (1.3, 2.6) mmol/L vs 1.4 (1.2, 1.8) mmol/L; 2.4 (1.8, 3.3) mmol/L vs 2.2 (1.7, 2.9) mmol/L; 3.3 (2.6, 4.5) mmol/L vs 3.1 (2.4, 3.9) mmol/L]. However, the levels of FPG and HbAlc in two groups were relatively comparable during the pregnancy. Even so, comparisons between the groups showed that the FPG level of OG was higher than that of NG in the 2^nd^ [(5.1 ± 0.5) mmol/L vs (4.9 ± 0.5) mmol/L, *P* < 0.001] and 3^rd^ trimester [(5.0 ± 0.6) mmol/L vs (4.9 ± 0.5) mmol/L, *P* < 0.05], while HbAlc level was only higher than that of NG in the 2^nd^ trimester [(5.1 ± 0.4) % vs (5.0 ± 0.4) %, *P* < 0.001].

### Relationship between biochemical indexes and birth weight

A simple linear regression analysis between each Z-score standardized index and birth weight was made (Table 2). We found that TG and FPG levels in the 1^st^ trimester and FPG level in the 3^rd^ trimester were positively correlated with birth weight. For each SD increase in TG and FPG level in the 1^st^ trimester and FPG level in the 3^rd^ trimester, birth weight increased by 89.0 g, 61.9 g and 58.9 g, respectively. However, there was a negative correlation between maternal ferritin level and birth weight in the 3^rd^ trimester, and birth weight decreased by 51.1 g for each SD increase in ferritin level. Then, the indexes with *P* < 0.2 in Table 2 were chosen for further analysis (Table 3**)**. After adjusting for maternal age, educational level, pre-pregnancy BMI, gestational age, gestational weight gain, parity and newborn sex, only the FPG level in the 3^rd^ trimester was positively correlated with birth weight, with birth weight increased by 44.9 g (14.0–75.7 g) for each SD increase in FPG level.

**Table 2:** Simple linear regression analyses between each* Z*-score standardized indexes and neonatal birth weight

	**The 1**^**st**^** trimester**			**The 2**^**nd**^** trimester**			**The 3**^**rd**^** trimester**		
	N	*β* per SD (95% *CI*)	*P*	N	*β* per SD (95% *CI*)	*P*	N	*β* per SD (95% *CI*)	*P*
Ferritin (μg/L)	334	5.9 (-44.3, 56.1)	0.817	483	6.2 (-34.9, 47.4)	0.766	357	-51.1 (-100.3, -1.8)	**0.042**
TC (mmol/L)	164	14.4 (-66.1, 94.9)	0.724	255	-20.2 (-75.7, 35.4)	0.475	405	-12.3 (-57.9, 33.2)	0.595
HDL-C (mmol/L)		-17.5 (-98.0, 63.0)	0.668		-20.2 (-75.7, 35.3)	0.474		-31.8 (-77.2, 13.7)	0.170
LDL-C (mmol/L)		26.5 (-54.4, 107.4)	0.519		-11.5 (-67.1, 44.1)	0.684		-17.2 (-62.9, 28.5)	0.460
TG (mmol/L)		89.0 (9.7, 168.4)	**0.028**		30.4 (-25.1, 85.8)	0.282		34.1 (-11.3, 79.5)	0.141
FPG (mmol/L)	287	61.9 (8.6, 115.3)	**0.023**	293	32.0 (-19.2, 83.2)	0.219	597	58.9 (22.0, 95.7)	**0.002**
HbAlc (%)	88	42.1 (-51.6, 135.9)	0.374	637	8.5 (-27.3, 44.2)	0.643	239	38.2 (-19.6, 96.0)	0.194

*TC* Total cholesterol, *HDL-C* High-density lipoprotein cholesterol, *LDL-C* Low-density lipoprotein cholesterol, *TG* Triglyceride, *FPG* Fasting plasma glucose

The biochemical variables were standardized by *Z*-score

**Table 3:** Beta coefficients (95% CI) for maternal ferritin, serum lipid and glucose level across pregnancy (per SD), in association with neonatal birth weight

	**N**	**Crude-** ***β*** ** per SD**	***P***	**Adjusted-*β***^**a**^** per SD**	***P***
**The 1**^**st**^** trimester**					
TG (mmol/L)	164	89.0 (9.7, 168.4)	**0.028**	55.4 (-10.2, 121.1)	0.097
FPG (mmol/L)	287	61.9 (8.6, 115.3)	**0.023**	35.8 (-7.3, 78.9)	0.103
**The 3**^**rd**^** trimester**					
Ferritin (μg/L)	357	-51.1 (-100.3, -1.8)	**0.042**	-12.1 (-53.5, 29.3)	0.564
HDL-C (mmol/L)	405	-31.8 (-77.2, 13.7)	0.170	-21.2 (-59.9, 17.5)	0.282
TG (mmol/L)	405	34.1 (-11.3, 79.5)	0.141	30.1 (-8.4, 68.5)	0.125
FPG (mmol/L)	597	58.9 (22.0, 95.7)	**0.002**	44.9 (14.0, 75.7)	**0.004**

a: Adjusted for maternal age, educational level, pre-pregnancy BMI, gestational age, gestational weight gain, parity and newborn sex

*TG* triglyceride, *FPG* Fasting plasma glucose, *HDL-C* High-density lipoprotein cholesterol

The biochemical variables were standardized by *Z*-score

### Relationship between FPG level in the 3^rd^ trimester and the risk of high birth weight

**Fig. 2:**
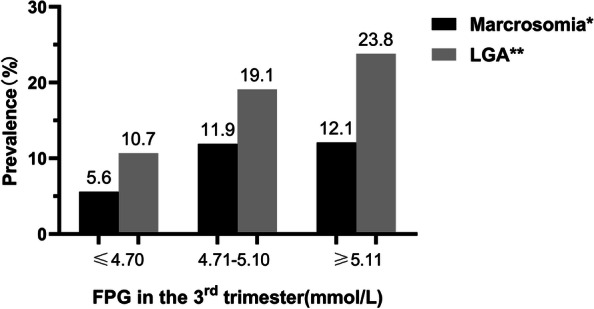
Prevalence of macrosomia and LGA newborns according to tertiles of FPG level in the 3.^rd^ trimester. FPG: fasting plasma glucose; LGA: large-for-gestational-age; *: *P* for trend = 0.004 < 0.05; **: *P* for trend < 0.001

Next, we divided the FPG level in the 3^rd^ trimester into tertiles and calculated the proportions of macrosomia and LGA in each category (Fig. 2). As expected, the prevalence of macrosomia and LGA increased with the increase of FPG levels (*P* for trend < 0.05), with 12.1% and 23.8% of marcrosomia and LGA, respectively in the 3^rd^ tertile. Logistic regression analyses results (Table 4) showed that the risk of macrosomia and LGA increased with the level of FPG in the 3^rd^ trimester (*P* for trend < 0.05). Even in Model 3 after adjusting for all confounders mentioned above, the risk of macrosomia and LGA increased by 1.44 and 1.50 times in the 3^rd^ tertile of FPG level compared to the 1^st^ tertile.

**Table 4:** Odds Ratios and 95% Confidence Interval for macrosomia and LGA according to tertiles of FPG level in the three trimesters

	**1**^**st**^** tertile** **(≤ 4.70 mmol/L)**	**2**^**nd**^** tertile** **(4.71–5.10 mmol/L)**	**3**^**rd**^** tertile** **(≥ 5.11 mmol/L)**	***P*** ** for trend**
**Macrosomia**				
Model 1	1.00 (Ref)	2.27 (1.08–4.81)	2.34 (1.12–4.89)	**0.030**
Model 2	1.00 (Ref)	2.42 (1.13–5.20)	2.33 (1.09–4.95)	**0.038**
Model 3	1.00 (Ref)	2.52 (1.14–5.57)	2.44 (1.12–5.33)	**0.035**
**LGA**				
Model 1	1.00 (Ref)	1.98 (1.11–3.52)	2.62 (1.50–4.56)	**0.001**
Model 2	1.00 (Ref)	2.05 (1.14–3.69)	2.58 (1.46–4.56)	**0.001**
Model 3	1.00 (Ref)	2.06 (1.13–3.76)	2.50 (1.40–4.47)	**0.002**

Model 1: unadjusted; Model 2: adjusted by maternal age, pre-pregnancy BMI, educational level, family history of diabetes and parity; Model 3: adjusted by maternal age, pre-pregnancy BMI, educational level, family history of diabetes and parity plus gestational age, gestational weight gain and newborn sex

*LGA* Large-for-gestational-age

## Discussion

In this study, we explored the trend changes of ferritin, serum lipid profile, plasma glucose and HbAlc levels in different pregnant trimesters in women with GDM with normal and high pre-pregnancy BMI and analyzed their effect on newborns’ birth weight. We found that regardless of pre-pregnancy BMI, the level of ferritin showed a downward trend during pregnancy whereas the levels of lipid profiles all showed upward trends while the levels of FPG remained relatively stable, although the average levels were generally higher in women who were overweight/obese before pregnancy. However, only FPG in the third trimester was positively associated with birth weight and the risk of macrosomia and LGA.

A large number of studies have shown that ferritin is closely related to the risk of GDM [8, 18–22]. The mechanism may be that the increase of serum ferritin induces the inflammatory process, which leads to the decrease of insulin secretion, the increase of insulin resistance and the disorder of liver function, and finally the decrease of muscle glucose uptake and the increase of gluconeogenesis, leading to the development of GDM [23, 24]. So we firstly analyzed the changes of ferritin levels in women with GDM during the whole pregnancy, and found that ferritin levels decreased in both women with GDM with normal and high pre-pregnancy BMI, which was consistent with the results of a randomized, double-blinded study of black women [25]. In addition, we also found that the ferritin levels of women with GDM who were overweight/obese before pregnancy were higher than those with normal pre-pregnancy BMI in the first and second trimester of pregnancy (Fig. 1). Previous studies have shown that obese and overweight adolescents have higher ferritin level than adolescents of normal weight [26, 27], possibly because inflammatory factors were activated during obesity and, accordingly, the iron regulating protein hepcidin was released as a defense mechanism, caused an increase in ferritin level [28]. Moreover, we did not find any effect of ferritin levels at all stages of pregnancy on birth weight after adjustment, which was similar to the opinions from previous studies [24].

Serum lipids are important indicators to reflect the health of the mother and fetus during pregnancy and dyslipidemia during pregnancy could increase the risk of adverse pregnancy outcomes such as GDM, preeclampsia and preterm delivery [7, 29]. The change of maternal lipid metabolism is a normal part of pregnancy. Fat accumulates in maternal tissue in the early stage of pregnancy and hyperlipidemia occurs in the third trimester of pregnancy [10]. But how is the situation in women with GDM? Ryckman et al. [30] once found that TG levels continued to increase throughout pregnancy in GDM patients. A previous study further revealed that changes in TG and TC levels were mainly caused by pre-pregnancy BMI [31]. In this study, we also found that the level of TC、HDL-C、LDL-C、TG increased during pregnancy in all women with GDM. And, the TG level of women with GDM with high pre-pregnancy BMI was higher than that of people with normal pre-pregnancy BMI. Since hypertriglyceridemia was considered to be one of the key driving factors of macrosomia [32], women with GDM with high BMI before pregnancy should be more vigilant against the occurrence of high birth weight. However, after adjusting for possible confounders, we found lipid levels at all stages of pregnancy had no effect on birth weight. This was inconsistent with the results of a multi-center Korean study in which a positive association between serum TG levels and birth weight in both the second and third trimester was found [33]. The main reason for the discrepancy may be that the population we studied was only women with GDM, which may affect the relationship between TG and birth weight.

Glucose metabolism is of particular concern throughout pregnancy and often played crucial role in the development of GDM. Previous studies showed that the mean HbA1c level decreased from the 1^st^ trimester until the 2^nd^ trimester, and increased towards the 3^rd^ trimester in women with pre-gestational diabetes [34], and there was a slight change in FPG level in the cohort of healthy pregnant women [35]. In this study, the levels of FPG and HbAlc of women with GDM remained in a relatively stable range during the whole pregnancy, because the glucose of women with GDM was controlled more strictly than that of normal women. Even so, further comparisons between the groups showed that the FPG level of OG was higher than that of NG in the 2^nd^ and 3^rd^ trimester, while HbAlc level was higher than that of NG in the 2^nd^ trimester. This is consistent with the finding that the values of OGTT in obese women were on the high side in the second trimester [36]. In addition, a study from Norway also suggested that women who were overweight/obese had significantly higher FPG in the 3^rd^ trimester than women with normal weight [35]. The results of multiple linear regression analysis showed that there was only a correlation between FPG in the 3^rd^ trimester and birth weight (Table 3). And the results of further exploration showed that high levels of FPG in the 3^rd^ trimester could increase the risk of macrosomia and LGA. The risk of macrosomia and LGA in GDM patients with FPG > 4.71 mmol/L in late pregnancy is more than twice as high as that in women with FPG ≤ 4.70 mmol/L based on the data of our study (Table 4), which means that women with GDM with normal and high pre-pregnancy BMI should pay special attention to FPG in the 3^rd^ trimester. However, a large cohort study of healthy pregnant women found that FPG in both 1^st^ and 3^rd^ trimester of pregnancy were associated with birth weight, and there was a negative correlation between HDL-C and birth weight in the 3^rd^ trimester [35], which is slightly different from the findings of this study. The reason may be the difference in study population or sample size and confounding factors, which still needs further study.

### Strengths and limitations

One of the major strengths of our study was that, to the best of our knowledge, this was the first time the associations of key clinical maternal biochemical indexes of three trimesters with neonatal birth weight were examined. Previous studies indicated that the level of maternal biochemical indexes, such as ferritin, serum lipid, plasma glucose and so on may have a predictive effect on neonatal birth weight [11–13]. However, majority of those studies only focused on a certain time point of pregnancy and it was hard to reveal the real effect on birth weight. Secondly, we did all analyses based on different pre-pregnancy BMI levels of subjects since the BMI before pregnancy has an important influence on the pregnancy outcome of women with GDM, including neonatal birth weight [37, 38]. However, some limitations of our study have to be considered. Firstly, this was a cross-sectional study needs to be further validated in conjunction with cohort studies. Based on our XAMC cohort, an ongoing observation study will prospectively provide more useful hints. Secondly, since women with GDM who were underweight before pregnancy were not included because of very small proportion, whether the conclusion can be applied to women with GDM with lower BMI before pregnancy is unknown.

## Conclusions

In women with GDM regardless of pre-pregnancy BMI, the level of ferritin decreased whist the levels of lipid profiles increased, and the levels of FPG remained relatively stable during the whole pregnancy. However, only FPG in the 3^rd^ trimester was an independent predictor of newborn birth weight, and that is associated with an increased risk of macrosomia and LGA. These observations highlight the importance of controlling the levels of FPG levels of women with GDM during pregnancy clinically.

## Data Availability

The datasets used and/or analyzed during the current study are available from the corresponding author on reasonable request.

## Abbreviations

GDM: Gestational diabetes mellitus
BMI: Body mass index
XAMC: Xi’an Longitudinal Mother–Child Cohort study
NG: Normal group
OG: Overweight/obesity group
FPG: Fasting plasma glucose
TG: Triglycerides
TC: Total cholesterol
HDL-C: High density cholesterol
LDL-C: Low density cholesterol
HbA1c: Glycosylated hemoglobin
OGTT: Oral glucose tolerance test
LGA: Large for gestational age
SGA: Small for gestational age
LBW: Low birth weight
OR: Odds ratio
CI: Confidence interval
